# Green algae *Ulva lactuca*-derived biochar-sulfur improves the adsorption of methylene blue from water

**DOI:** 10.1038/s41598-024-61868-9

**Published:** 2024-05-21

**Authors:** Amany G. M. Shoaib, Huu-Tap Van, Dinh-Trinh Tran, Amany El Sikaily, Mohamed A. Hassaan, Ahmed El Nemr

**Affiliations:** 1 Environment Division, National Institute of Oceanography and Fisheries (NIOF), Kayet Bey, Elanfoushy, Alexandria, Egypt; 2 Center for Advanced Technology Development, Thai Nguyen University, Tan Thinh Ward, Thai Nguyen City, 25000 Vietnam; 3 VNU Key Lab. of Advanced Materials for Green Growth, University of Science, Vietnam National University, No. 19 Le Thanh Tong Street, Hoan Kiem, Hanoi, 120000 Vietnam

**Keywords:** Methylene blue adsorption, Sulphuric acid-modified biochar, Green algae *Ulva lactuca*, Dye removal, Chemical engineering, Environmental chemistry

## Abstract

The present investigation explores the efficacy of green algae *Ulva lactuca* biochar-sulfur (GABS) modified with H_2_SO_4_ and NaHCO_3_ in adsorbing methylene blue (MB) dye from aqueous solutions. The impact of solution pH, contact duration, GABS dosage, and initial MB dye concentration on the adsorption process are all methodically investigated in this work. To obtain a thorough understanding of the adsorption dynamics, the study makes use of several kinetic models, including pseudo-first order and pseudo-second order models, in addition to isotherm models like Langmuir, Freundlich, Tempkin, and Dubinin–Radushkevich. The findings of the study reveal that the adsorption capacity at equilibrium (*q*_e_) reaches 303.78 mg/g for a GABS dose of 0.5 g/L and an initial MB dye concentration of 200 mg/L. Notably, the Langmuir isotherm model consistently fits the experimental data across different GABS doses, suggesting homogeneous adsorption onto a monolayer surface. The potential of GABS as an efficient adsorbent for the extraction of MB dye from aqueous solutions is highlighted by this discovery. The study’s use of kinetic and isotherm models provides a robust framework for understanding the intricacies of MB adsorption onto GABS. By elucidating the impact of various variables on the adsorption process, the research contributes valuable insights that can inform the design of efficient wastewater treatment solutions. The comprehensive analysis presented in this study serves as a solid foundation for further research and development in the field of adsorption-based water treatment technologies.

## Introduction

The discharge of dangerous chemicals and dyes into textile wastewater poses a serious threat to the environment. Numerous treatment approaches have been investigated to tackle this problem^1–3^, which covered the oxidation of reactive dyes with in situ electro-generated active chlorine for wastewater treatment in the textile dyeing industry. They highlighted the sustainability challenges textile industries face in managing solid and liquid waste, leading to environmental pollution^4^. Sarkar et al.^5^ emphasized the significant contribution of the textile industry to industrial water pollution, with 17–20% attributed to dyeing treatments. In their evaluation of electrocoagulation technology, Naje et al.^6^ addressed the poor rate of pollutant removal and the production of a significant amount of sludge in traditional coagulation procedures. This technology is used to treat textile wastewater.

Moreover, Halepoto et al.^7^ discussed the use of anaerobic and aerobic processes for treating high and low chemical oxygen demand (COD) textile wastewater, respectively. A staged forward osmosis technique was proposed by Kim et al.^8^ and Li et al.^9^ for the simultaneous desalination and concentration of textile wastewater. They recognised water pollution as a major environmental problem related to the textile industry and emphasized using the best possible technology to reduce air and water pollutants from textile dyeing and finishing. Furthermore, Bazrafshan et al.^10^ studied textile wastewater treatment using electrocoagulation and compared the performances of various electrode connection modes.

Textile wastewater treatment is a critical environmental concern, and sustainable techniques, such as adsorption engineering, have gained significant attention^11–14^. Among several physical and chemical wastewater treatment methods that have been used in recent years to remove dyes^14^, adsorption is one of the most effective techniques that has been successfully used to remove dyes from industrial wastewater. High adsorption capacity materials have the potential to be used in adsorption procedures that remove a variety of contaminants from textile effluent, such as organic compounds, heavy metals, and dyes^4,15,16^. These materials can vary from traditional activated carbon to innovative options like biochar or modified clays^17–19^. The advantages of adsorption engineering in treating textile wastewater lie in its efficiency in removing various pollutants^12^. This includes effectively fractioning dyes and salts using ultrafiltration membranes^20^. Besides, the use of materials like red mud, eggshells, and biochar has been explored for the removal of specific dyes from wastewater, highlighting the versatility of adsorption processes^21–23^. The potential of biochar for removing organic and inorganic pollutants from industrial effluents has been highlighted, emphasizing its versatility and effectiveness in wastewater treatment^24–27^.

Furthermore, the use of engineered biochar, incorporating appropriate magnetic media, has been explored for effective separation from the aqueous phase, enhancing its potential for practical applications in wastewater treatment^28–31^. The application of biochar for water and wastewater treatment has been extensively reviewed, showcasing its high sorption capacity for various contaminants, including hydroquinone^32^. Researchers have also looked at using biochar in biofiltration systems to remove pathogens, nutrients, and pharmaceutical and personal care items from wastewater. This shows that biochar can remove a variety of contaminants^33^. The influence of nitrogen modification on adsorption capacity was demonstrated by the improved phenol adsorption displayed by the nitrogen-doped hierarchical porous biochar made from maize stalks^34^. Additionally, modification methods, including HNO_3_, NaOH, and Na_2_S, have been shown to enhance the adsorption capacity of biochar for Mn(II) removal^35^. Additionally, the excellent adsorption characteristics of cationic dyes have been proven through the use of calcium-rich biochars generated from wasted mushroom substrates, indicating the possibility of particular modifications for targeted adsorption applications^36^.

The use of biochar derived from green algae *Ulva lactuca* (*U. lactuca*) for the adsorption of pollutants from aqueous solutions is an innovative and eco-friendly approach in the field of environmental remediation. Green algae, as source material for biochar, represents a sustainable and renewable resource, as it can be cultivated in various water bodies, including those unsuitable for other uses^37–42^. Algae-based biochar, especially when modified, can have a high surface area and porosity, making it highly effective for adsorbing pollutants, including heavy metals, dyes, and organic compounds from wastewater^28,43–45^. Moreover, it emphasizes that the presence of N-functional groups in algae-based biochar improved its surface chemistry and adsorption capacity^46^. These findings underscore the potential of modified biochar from green algae for efficient adsorption of contaminants.

The investigation into utilizing modified biochar derived from green algae *U. lactuca* for the adsorption of Methylene Blue from aqueous solutions constitutes a significant and innovative contribution to the wastewater treatment domain. This study fills a critical void in existing literature, predominantly concentrating on conventional and frequently less sustainable approaches to treating textile wastewater. The novelty of the present research lies in its examination of a sustainable and effective alternative utilizing biochar derived from *U. lactuca*. In contrast to traditional adsorbents, green algae biochar presents a renewable, cost-efficient, and environmentally friendly solution. The modification of this biochar to augment its adsorption capacity, particularly for methylene Blue, showcases a pioneering strategy to target specific pollutants commonly found in textile wastewater. Moreover, the utilization of green algae *U. lactuca*, an easily accessible and underutilized biomass, for biochar production not only offers an efficient remedy for wastewater treatment but also contributes to managing algal blooms and carbon sequestration. This research addresses the gap by presenting a pragmatic, scalable, and environmentally sustainable approach, underscoring the potential of biochar modifications to fulfil specific industrial wastewater treatment requirements. This facet has not been extensively explored in prior studies.

This study aims to employ biochar derived from the green algae *U. lactuca* using concentrated H_2_SO_4_ and NaHCO_3_ to adsorb MB dye from aqueous solutions. The study will evaluate factors such as initial concentration, contact time, and pH through batch experiments. Furthermore, the application of adsorption models will be utilized to comprehend the mechanism and kinetics of the adsorption process. This approach represents an innovative and sustainable method in industrial wastewater treatment, aiming to mitigate detrimental.

## Materials and methods

### Chemicals and equipment

**Figure 1:**
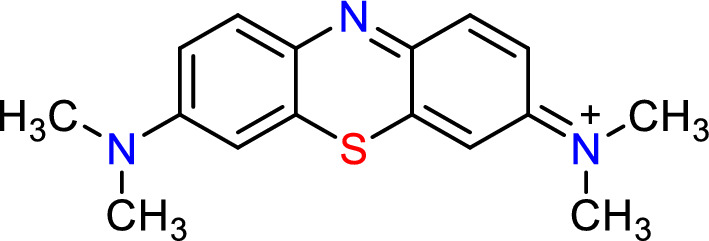
Methylene blue dye chemical structure.

Sulfuric acid (H_2_SO_4_ (99%), Mwt = 98.079 g), Sodium bicarbonate NaHCO_3_ (Mwt = 84 g), Sodium Hydroxide (NaOH, Mwt = 39.997 g), Hydrogen Chloride (HCl , Mwt = 36.46 g). MB dye (basic blue 9; C.I. 52,015, M.F. = C_16_H_18_N_3_ClSxH_2_O, λ_max_ = 665 nm, M.W. = 319.86 g/mol, 97% dye content) (Fig. 1) was acquired from Honeywell Riedel-de Haën AG, Seelze-Hannover, a JENCO-6173 pH metre, Germany. A JSOS-500 shaker, and a digital UV/Visible spectrophotometer (PG instrument model T80 Doublebeam UV/visible spectrophotometer, United Kingdom, with glass cells and a 1 cm optical path) were used in this work.

### Sampling

Green algae *U. lactuca* were harvested from the coastal region of Alexandria, cleaned with tap and distilled water, and then allowed to air dry for one night at room temperature before being baked for seventy-five hours at 120 °C. then used the automatic mixer to compress it into a fine powder. Before use, the green powder was put through a 100-mesh screen. The obtained *U. lactuca* samples have the following characteristics: Bulk density of 0.8179 g/mL, Ash content of 35.12%, and moisture content of 15.3%.

### Preparation of stock solution

A stock solution containing 1000 mg/L of MB dye was prepared by dissolving 1.0 g in 1000 mL distilled water (DW). Once the concentrations needed for the standard calibration curve and adsorption studies were determined, this stock solution was diluted further.

### Preparation of GABS

300 g of dried *U. lactuca* powder was added to 600 mL of concentrated H_2_SO_4_, followed by the addition of 70 mL of DW. The system was then refluxed for 4 h at 300 °C, filtered, and then washed with DW and 1% NaHCO_3_ solution to eliminate any residual acid until the filtrate became neutral. Finally, ethanol was added to remove any impurities and any residual deep organic materials and the mixture was dried for 24 h at 120 °C to produce 122 g (40.67% yield) of the targeted biochar. This type of carbon is called sulfonated biochar carbon GABS.

### Batch adsorption procedure

A series of adsorption batch equilibrium experiments were conducted to examine the biochar’s ability to remove MB dye including the impact of initial adsorbate concentration, contact duration, and pH on the adsorption efficiency^47,48^. Batch adsorption studies were studied in a set of Erlenmeyer flasks with 100 mL of varied MB dye solution concentrations (100, 125,150,175 and 200 mg/L) and varying doses of GABS (0.5, 0.75, 1.0, 1.25, and 1.5 g/L) at 25 °C. Up to a certain point, an isothermal shaker operating at a set 200 rpm and constant temperature was employed.

An initial concentration (100–200 mg/L for MB dye) was created to investigate the effects of contact duration and initial adsorbate concentration on the adsorption uptake. The impact of solution pH on the adsorption process was investigated by changing the starting pH of the solutions from 2 to 12. Furthermore, by fitting the equilibrium data to the Langmuir, Freundlich, and Temkin models, respectively, isotherm investigations were conducted for adsorption. The kinetics of the adsorption process were investigated in the batch kinetic studies using the pseudo-first-order (PFO), pseudo-second-order (PSO), Elovich, and intraparticle diffusion methods^47,49,50^. Upon utilising the aforementioned models to depict the kinetic data, each experiment’s *q*_cal_, *q*_exp_, slope, and intercept were determined, along with the least-squares correlation coefficient (*R*^2^). Equation (1) was used to predict the adsorption capacity at equilibrium (*q*_e_).

1 $$ q_{e} = \frac{{\left( {C_{0} - C_{e} } \right) V}}{m} $$

where the ability of the adsorbent to adsorb MB dye from a solution at a specific time is expressed as the adsorption capacity (*q*_e_) (mg/g). The initial concentration of MB dye is *C*_0_ (mg/L), and the concentration of MB dye that remains after a given amount of time is *C*_e_ (mg/L). You may use Eq. (2) to find the percentage of MB dye removed from water.

2 $$ Removal \% = \frac{{\left( {C_{0} - C_{e} } \right) }}{{C_{0} }}{ } \times 100 $$

### Determination of point of zero charge pH_ZPC_

To determine the pH_ZPC_ value of prepared activated carbons, 0.1 g of activated carbon was introduced into 50 mL of 0.1 M NaNO_3_ solution with an initial pH range of 3–10 and was shaken for 24 h. After that, the sample was separated from the solution and the solution equilibrium pH values were measured and the pH_ZPC_ value was obtained.

### Characterization

The characteristics of GABS were examined using FTIR spectroscopy, proximate analysis, XRD, EDX, SEM, and surface area analysis. The dry GABS powder’s X-ray diffraction pattern (XRD) was obtained at an angle of 2*θ* using an X-ray diffractometer (Ulitama IV, Rigaku, Tokyo, Japan). Using a field emission scanning electron microscopy (SEM; QUALITY 250) with an acceleration voltage of 15.0 kV, the size and shape of biochar were examined. EDX was used to determine the materials’ elemental concentrations of C, N, and O. Using the multipoint N_2_ adsorption Brunauer–Emmett–Teller (BET) technique (BELSORP-Mini II, BEL Japan, Inc.), specific surface area, pore size, and pore volume were determined. The functional groups on the surface of GABS were recognized by the Fourier transfer infrared (FTIR) spectrometer (VERTEX70 and ATR unit model V-100) in the region of 400–4000 cm^−1^. Using a thermogravimetric analyzer (TGA), approximate analyses of the GABS were conducted (SDT650-Simultaneous Thermal Analyzer apparatus). The percentages of moisture, volatile matter, fixed carbon, and ash were computed at 100% based on the TGA results. To assess the moisture content, GABS was heated in N_2_ gas from room temperature to 110 °C until dehydration was achieved. GABS underwent breakdown at a temperature rise rate of 5 °C/min, with a temperature range of 25–1000 °C^25,51,52^.

## Result and discussion

### Characteristics of GABS

**Figure 2:**
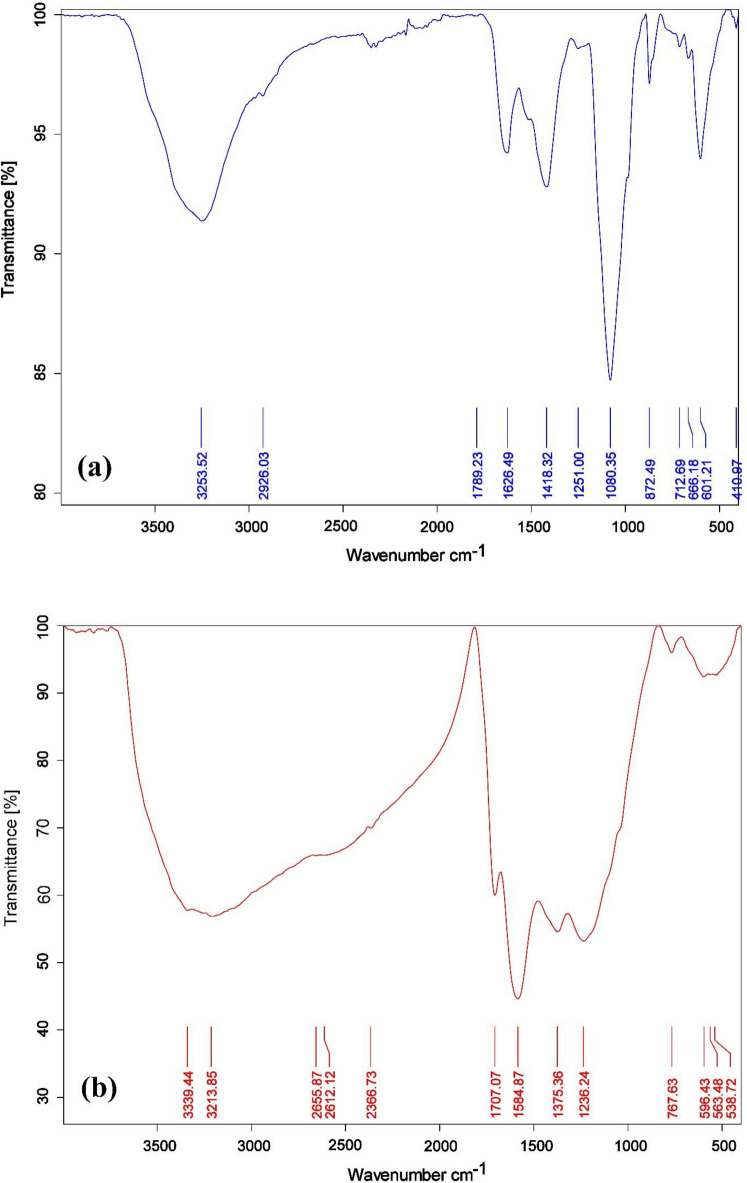
FTIR graphs of (**a**) green algae *U. lactuca* and (**b**) GABS.

The functional groups existing on the surface of Green algae *U. lactuca* and the resulting GABS adsorbent were identified Using FTIR spectroscopy. The FTIR diagrams of the *U. lactuca* and GABS were compared (Fig. 2a,b). The materials’ FTIR spectra reveal modifications to their functional groups. The stretching oscillation of the O–H present in the *U. lactuca* and GABS is shown by the broadband at 3253.52 cm^–1^ (Fig. 2a) for *U. lactuca* and 3339.40 and 3213.85 cm^–1^ (Fig. 2b) for GABS. The strong adsorption peak at 2926.03 cm^–1^ (Fig. 2a) suggests the existence of –CH_2_ stretching groups in *U. lactuca*. According to Fig. 2b, these groups were broadband at 2655.87 and 2612.12 cm^–1^ in GABS. The tiny adsorption band at 1789.27 cm^–1^ is caused by the C=O stretching of the anhydride groups in the *U. lactuca* (Fig. 2a). Later on, at 1707.07 cm^–1^, this band changed into a carboxyl group in GABS (Fig. 2). However, when GABS was compared to raw *U. lactuca*, the strength increased to 1707.07 cm^–1^, suggesting that sulphoric acid treatment may enhance the carbonyl (C=O) group ^47^. The bands located at 1626.49 cm^–1^ indicate that the C=O stretching oscillation of the *β*-ketone was almost nonexistent in the *U. lactuca* (Fig. 2a). With high intensity, this oscillation moved to 1584.87 cm^–1^ in GABS; it might be a stretching vibration of –C=C– in GABS (Fig. 2b). The peaks at 1418.32–1251.00 cm^–1^ are indicative of the *U. lactuca* ‘ C–O functional group (Fig. 2a). The band at 1375.36 and 1236.24 cm^–1^ in GABS, which showed the sulfonyl group (SO) stretching vibration, took the position of this group (Fig. 2b). Additionally, the development of broad peak covered the area between 1236.00 and 940.00 cm^–1^ was produced by the dehydration process with H_2_SO_4_. These peaks resulted from the production of –SO_3_H and SO groups in GSBS. These bands show that the *U. lactuca* treatment with H_2_SO_4_ results in the creation of the GSBS. In contrast to GSBS, which had a faint band at 767.63 cm^–1^, *U. lactuca* displayed a more pronounced increase in the –C–O–C– asymmetric stretching functional group at 1080.35 cm^–1^ (Fig. 2a)^25,53–55^.

**Figure 3:**
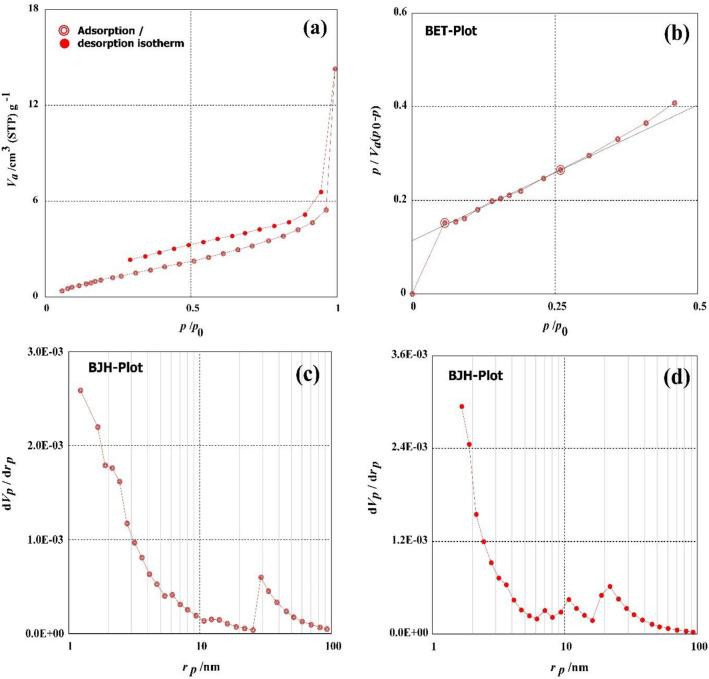
(**a**) Diagram of N_2_ adsorption–desorption, (**b**) diagram of the BET, (**c**) diagram of the BJH adsorption, (**d**) diagram of the BJH desorption of the GABS.

The N_2_ adsorption–desorption isotherm of the GABS was examined to determine how H_2_SO_4_ altered the surface properties of the GABS. The BET and BJH approaches were used to determine the specific surface area and mesopore area, respectively. The textural characteristics of GABS, such as the average pore diameter, monolayer volume, mass of mesopores, mesopore area, total volume of pores, and mesopore distribution peak, are displayed in Fig. 3. At 6.26 m^2^/g, the GABS has a comparatively small BET-specific surface area. The monolayer volume value of DABS was 1.4389 cm^3^ (STP) g^–1^. The overall volume of GABS is 2.0309 × 10^–2^ cm^3^/g. The overall pore volume of DRB-SO was 1.8975 × 10^–2^ cm^–1^, with mean pore sizes of 12.971 nm. The mesopore distribution peak value, meso surface area peak value, and mesopore volume peak values of the GABS adsorption study were determined to be 1.22 nm, 2.1338 × 10^–2^ cm^3^/g, and 7.0477 m^2^/g, respectively. In the GABS study of adsorption, the mesopore volume, meso surface area, and mesopore distribution peak values were determined to be 4.3323 m^2^/g, 1.8970 × 10^–2^ cm^3^/g, and 1.66 nm, respectively.

**Figure 4:**
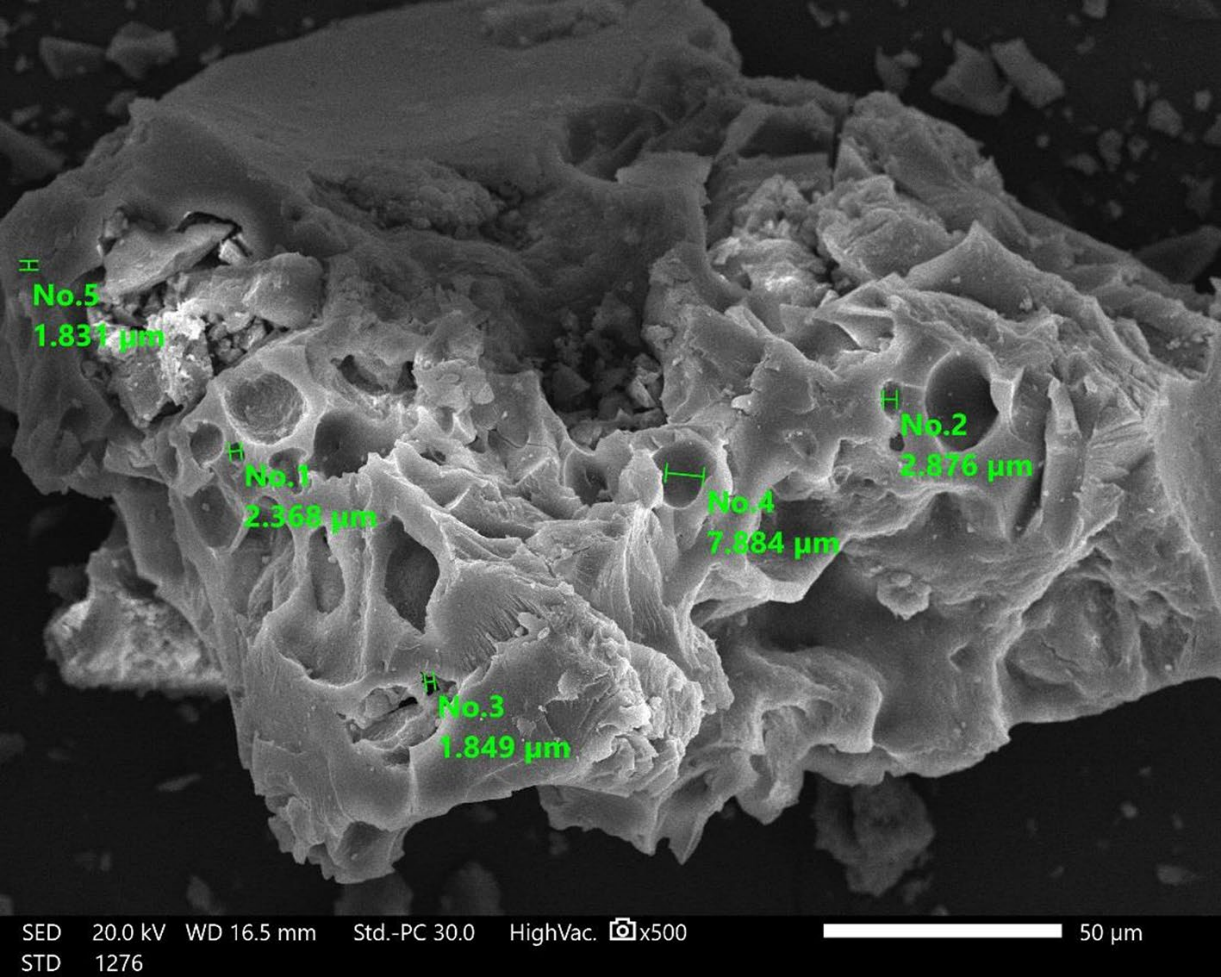
SEM image of GABS using high vacuum SEM at magnification × 500 and 15.0 kV.

Figure 4 displays SEM images of the GABS, demonstrating its purity and lack of contaminants. After the strong sulfuric acid treatment, the pore structure of the GABS remained intact. The particle size distribution shows that the particle sizes were within the range of 1800–7884 nm and the determined average particle size distribution of the GABS was 4241 ± 1.65 nm.

Using scattered X-ray spectrometry (EDX), the chemical composition of the GABS adsorbent was investigated. Table 1 displays the percentage of each element. It shows that, in addition to carbon mass percentage, which accounts for 53.59% of the sample, there are approximately 41.70 and 1.29% of oxygen and sulphur, respectively.

**Table 1:** EDX results of prepared GABS.

Elements	GABS	
	Mass%	Atom%
C	53.59 ± 0.30	62.02 ± 0.34
O	41.70 ± 0.61	36.24 ± 0.53
S	1.29 ± 0.06	0.56 ± 0.02
Ca	3.42 ± 0.11	1.19 ± 0.04
Total	100.00	100.00

**Figure 5:**
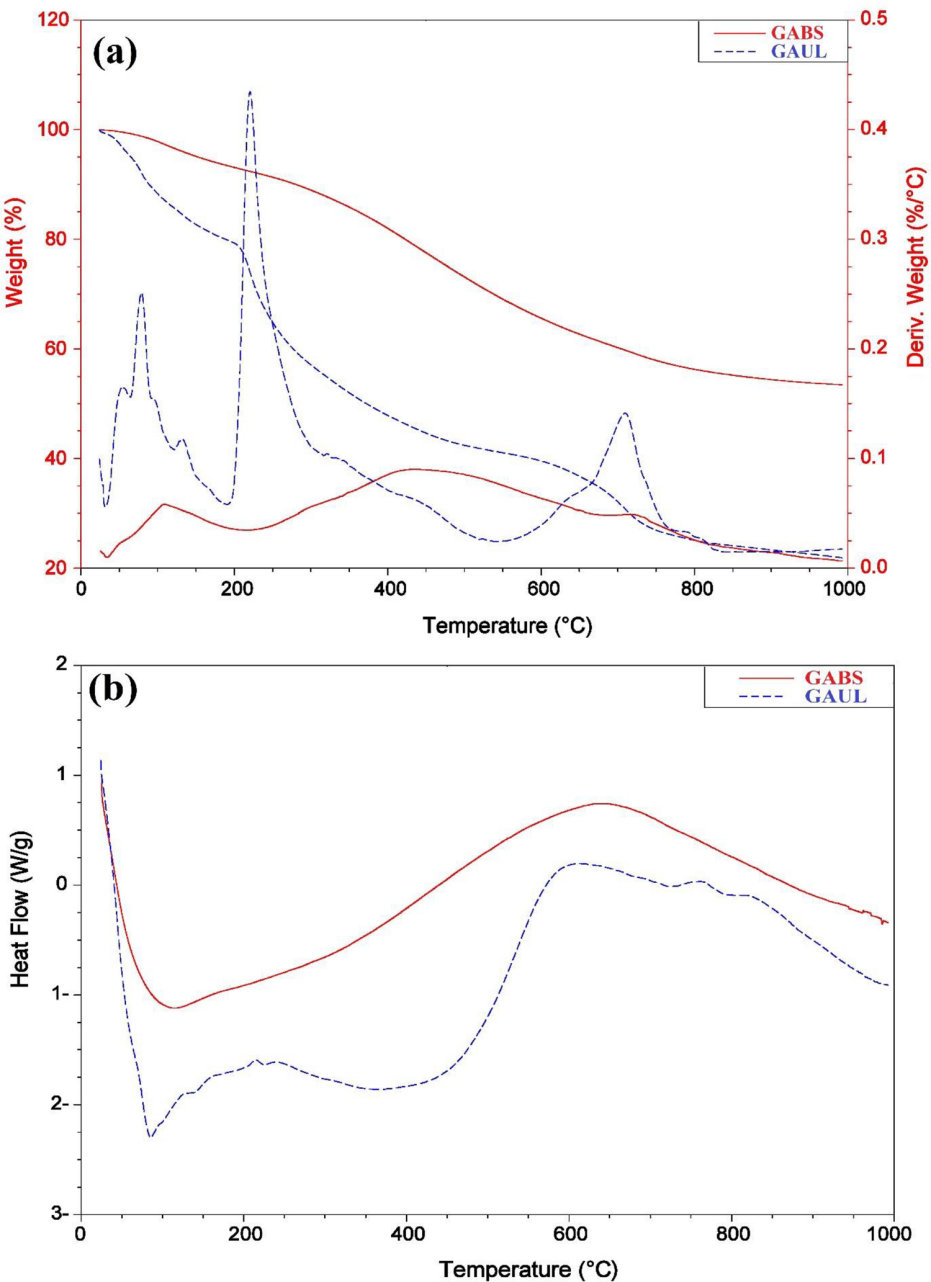
(**a**) Diagrams of TGA and TDA, and (**b**) diagram of DSC of the *U. lactuca* and GABS.

Thermal gravimetric analysis (TGA) was used to evaluate the effects of structural differences on the degradation behaviour and operating temperature of the raw *U. lactuca* and GABS samples. The materials were cooked between 50 and 1000 °C in a N_2_ environment. The analytical curves for TGA, differential thermal analysis (DTA), and differential scanning calorimetry (DSC) for *U. lactuca* and GABS adsorbent are shown in Fig. 5. The evaporation of water in the raw *U. lactuca* and GABS was the source of the first weight drop, which peaked before 200 °C. As the temperature increased over 200 °C, numerous acidic oxygen functional groups broke down, causing raw *U. lactuca* and GABS to lose weight. *U. lactuca* shows three weight losses at temperatures between 25 and 200, 200–550 and 550–950 °C with a total weight loss of 78.01%, while GABS shows also three weight losses at temperatures between 25 and 200, 200–700 and 700–950 °C with total weight loss of 46.52%, which explains the stability of GABS compared to the raw *U. lactuca* (Fig. 5a). Because of the breakdown of carbon in biomass, the TGA curves of GABS and *U. lactuca* converged at temperatures greater than 433 °C and 220 °C, respectively.

The DTA graph of raw *U. lactuca* and GABS is illustrated in Fig. 5a. The DTA curve of the raw *U. lactuca* peaked at three temperature points (*T*_*f*_, 78.97, 220.44 and 709.64 °C), and the curve of GABS peaked at three temperature points (*T*_*f*_, 108.16, 433.27 and 725.52 °C) (Fig. 5a). The DTA curve demonstrating the production of GABS adsorbents from raw *U. lactuca* indicates that the dehydration of raw *U. lactuca* yielded three distinct degradation bands. The GABS degradation bands occurred at a higher temperature after treatment with 90% H_2_SO_4_, demonstrating that the degree of degradation was intensely affected by treatment with H_2_SO_4_.

DSC may be used to compare materials by using thermal transitions or glass transitions. Figure 5b depicts the DSC graph of raw *U. lactuca* and GABS. The glass transition temperatures (*T*_g_) of *U. lactuca* occurred at 116.53, 145.82, and 170.69 °C, while GABS displays *T*_g_ values at 335.11, 353.26, and 363.76 °C. Higher transitional temperatures transformed the grains into more crystalline forms that were more resistant to gelatin breakdown and had better structural integrity.

**Figure 6:**
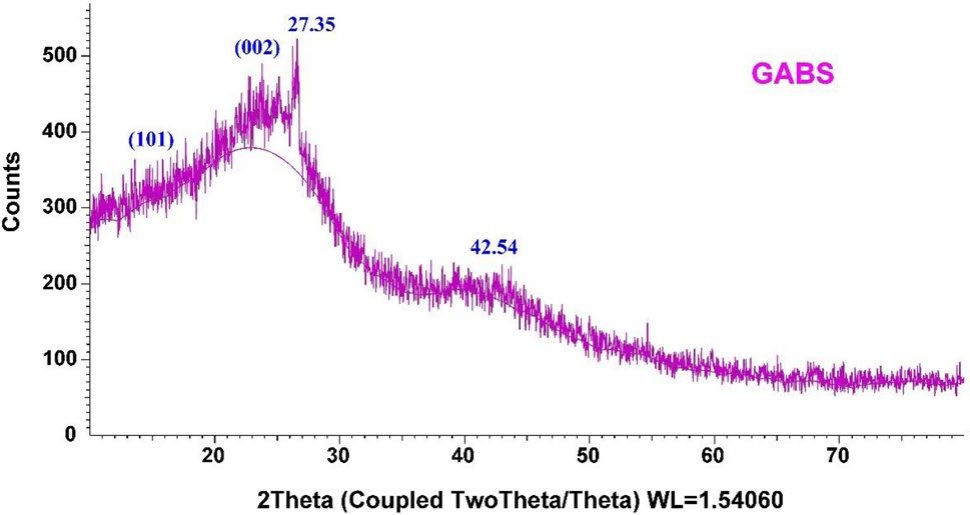
XRD graph of fabricated GABS biochar.

Figure 6 displays the results of the GABS XRD, which reveal an amorphous carbon structure with aromatic sheets orientated randomly. A broad peak is identified as the C (002) diffraction peak in the region of 2*Ɵ* ~ 24.9, whereas a minor peak is situated at 2*Ɵ* = 42.54. About 2*Ɵ* ~ 15.4 (101) notable cellulosic peaks is a small peak. This might indicate several inorganic substances, namely quartz and albite (a mineral that contains plagioclase feldspar)^56,57^.

### Solution pH effect on MB dye adsorption

**Figure 7:**
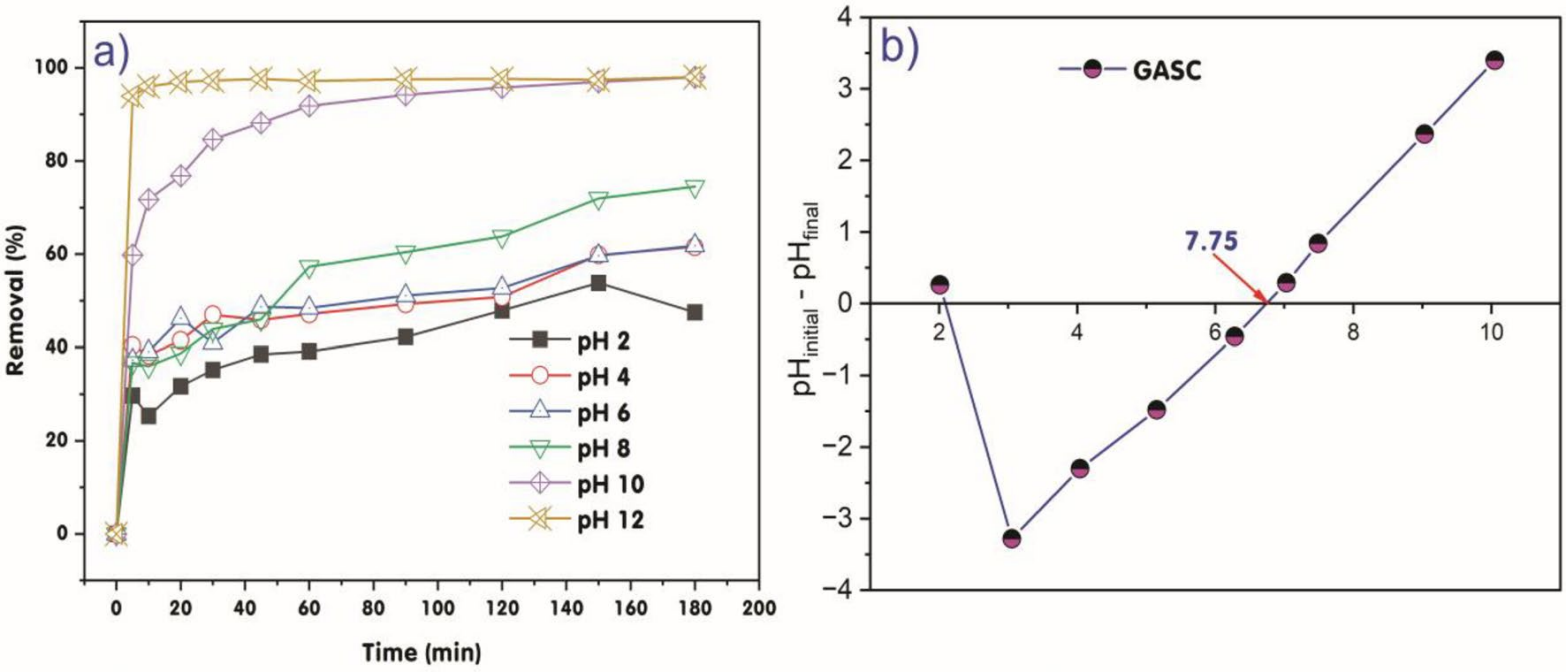
(**a**) The influence of pH values on the removal of MB dye through adsorption. The experimental conditions included a GABS dose of 1.0 g/L, initial MB dye *C*_0_ of 200 mg/L, temperature of 25 ± 2 °C, and contact time of 180 min, (**b**) pH_PZC_ analysis of GABS.

Upon analysis of the data provided in the study on the elimination efficiency of methylene blue (MB) dye using GABS under different pH conditions over time (Fig. 7a), several critical observations can be drawn. The general trend indicates that as the solution pH increased from 2 to 12, the adsorption efficiency of MB dye by GABS gradually increased over time, ranging from 5 to 180 min. Initially, at time *t* = 0, the removal efficiency of MB dye by GABS was 0% across all pH levels. However, as the time of contact increased, the removal efficiency of MB dye exhibited a significant dependence on both the contact time and the solution pH. Notably, at a lower pH of 2, the removal efficiency started at 29.69% after 5 min and peaked at 53.80% at 150 min before experiencing a slight decline to 47.49% at 180 min. This initial increase, followed by a decline, could suggest a saturation of the adsorption sites on GABS or possible desorption at extended contact times. As the pH increased to 4 and 6, there was a similar pattern of increased efficiency over time, with the highest removal efficiencies being 61.57% at pH 4 and 61.83% at pH 6, both observed at the 180-min mark. This indicates that the GABS has a relatively consistent adsorption capacity in the slightly acidic to neutral pH range.

Interestingly, the removal efficiency exhibited a distinct trend at higher pH values. At pH 10 and 12, there was a rapid increase in the removal efficiency within the first 10 min, reaching 71.72% and 95.97%, respectively. Moreover, at pH 12, the efficiency swiftly escalated to above 93.90% within 5 min. and plateaued around 98.00% from 150 min onward. Under these conditions, the removal efficiency of MB dye by GABS was optimal, reaching 95.97% after 150 min of adsorption at pH 12. This could be indicative of the GABS having a more favourable adsorption interaction with MB in a highly alkaline environment, possibly due to changes in the GABS’s surface charge or the MB dye molecules’ ionization state^55,58,59^.

The observed trend of increasing methylene blue (MB) dye removal efficiency by granular activated carbon derived from sugarcane (GABS) with increasing pH from 2 to 12 over time aligns with the general understanding of adsorption processes. The literature provides insights into the adsorption properties of various carbon-based materials, shedding light on the mechanisms underlying the observed trend. Shrestha et al.^60^ discussed the adsorption properties of rice husk-derived carbon materials, including excellent methylene blue adsorption properties, which can provide insights into the adsorption behaviour observed in the study. Moeinian and Mehdinia^61^ focused on removing MB dye from aqueous solutions using rice husk silica adsorbent, offering valuable information on adsorption processes. Furthermore, Kouassi et al.^62^ explored the removal of MB dye from industrial effluents using corncob-activated carbon, providing insights into the adsorption behaviour of activated carbon. Ndi Nsami and Ketcha Mbadcam^63^ also discussed the adsorption efficiency of chemically prepared activated carbon from cola nut shells for MB dye, offering further insights into the adsorption processes. These references collectively contribute to understanding GABS’s observed pH-dependent adsorption behaviour towards MB dye, providing a comprehensive perspective on the underlying mechanisms driving the observed trends.

The graph in Fig. 7b illustrates the pH_PZC_ (point of zero charge) values of GABS, which is determined to be 7.75. When the solution pH exceeds the pH_PZC_ of the adsorbent, the surface charge of the adsorbent becomes negative. The MB dye is classified as cationic, indicating a positive charge in solution^64,65^. At lower pH values, such as pH 2, the positive charge on the adsorbent’s surface results in electrostatic repulsion between the dye molecules and the adsorbent, leading to reduced adsorption of MB dye. Conversely, as the pH surpasses the isoelectric point (up to pH 12 in this instance), the surface of GABS becomes increasingly negatively charged due to the deprotonation of acidic functional groups. There is an electrostatic attraction between the positively charged MB dye molecules and the adsorbent, facilitating the adsorption of MB dye onto the surface of GABS. The transition from a positive to a negative charge on the adsorbent surface as the pH increases from a low value (e.g., pH 2) reduces electrostatic repulsion. Eventually, it leads to electrostatic attraction as the pH exceeds the isoelectric point (7.75) and rises. Consequently, the adsorption capacity for MB dye on GABS increases with rising pH, reaching optimal levels when the pH is well above the isoelectric point, as GABS’s highly negative surface charge favours the adsorption of the cationic dye molecules. This phenomenon is consistent with the principles of surface charge and electrostatic interactions in the context of adsorption processes.

### Contact time impact on MB dye adsorption

**Figure 8:**
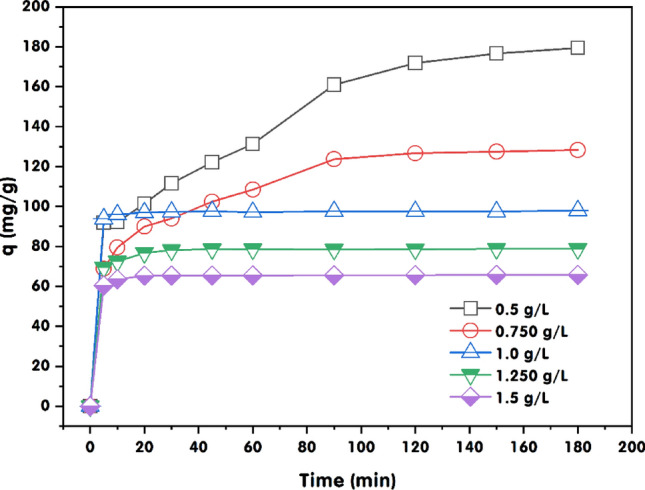
Contact time impact on MB dye adsorption by GABS. The experimental conditions included a pH of 12, *C*_0_ of MB dye of 200 mg/L, and temperature of 25 ± 2 °C.

Analyzing the provided dataset on GABS’s MB dye adsorption capacity across different adsorbent concentrations over time reveals several pertinent aspects regarding the adsorption kinetics and equilibrium states. The data from Fig. 8 demonstrates the rapid uptake of MB dye by GABS over time, indicating a typical initial phase of the adsorption process characterized by the abundance of available active sites. The influence of adsorbent concentration on the adsorption capacity is pronounced, with lower concentrations leading to a quick escalation in MB dye adsorption capacity, reaching 91.91 mg/g within 5 min and continuing to rise to a maximum of 179.41 mg/g at 180 min. This suggests that at lower adsorbent concentrations, the ratio of available adsorption sites to MB molecules is high, allowing more excellent individual adsorbent saturation. However, as the dose of GABS increases, the initial adsorption capacity decreases, indicating a dilution effect or fewer available adsorption sites per unit of adsorbent due to aggregation or reduced surface area exposure. Despite this, all concentrations of GABS approach a similar adsorption capacity value at the equilibrium state after 180 min, with the 1.0 g/L concentration showing a slightly higher equilibrium capacity of 98.00 mg/g compared to the others. The trend of adsorption capacity with increasing GABS dose does not strictly follow a linear relationship, suggesting diminishing returns as the concentration increases, possibly due to the overlapping of adsorption sites and the formation of aggregates at higher concentrations, which decreases the effective surface area available for adsorption.

The equilibrium adsorption capacity of MB by GABS increased gradually over time and reached saturation after 90–120 min with GABS dose increasing from 0.5 to 0.75 g/L. However, the saturation state was achieved after 20 min of adsorption for GABS concentrations ranging from 1.0 to 1.5 g/L, with a stable adsorption capacity of 171 mg/g observed for the 0.5 g/L GABS dos after 120 min. The observed increase in the adsorption capacity of MB dye by GABS over time, reaching saturation after varying durations at different GABS concentrations, also aligned with the findings of several relevant studies, for instance, demonstrated that the adsorption performance of cationic dyes, such as MB dye, by activated carbon modified with surfactants significantly influences the adsorption capacity^66^. This behaviour aligns with the findings of those who discussed MB dye adsorption and its complexes with montmorillonite, providing insights into the adsorption behaviour of cationic dyes^67^. The study demonstrated the selective adsorption of cationic dyes, offering further insights into the adsorption behaviour of cationic dyes^68^. Also, the work of Yamaguchi et al.^69^ examined the adsorption of the cationic dye MB dye, providing valuable insights into the adsorption behaviour of cationic dyes. Furthermore, studies on the creation of MoO_3_ nanowire-based membrane devices for the selective adsorption of cationic dyes from aqueous solutions are pertinent because they shed light on the process of selective adsorption, which advances our knowledge of the adsorption behaviour of cationic dyes^70^.

The reduction in MB dye adsorption capacity and the rapid attainment of saturation with increasing the adsorbent material (GABS) ratio can be attributed to several critical factors related to the adsorption process and the system’s physical characteristics. Firstly, adsorption is contingent on the availability of surface area for the adsorbate (MB dye) to interact with the adsorbent material (GABS). An increase in the ratio of adsorbent material augments the available surface area for the adsorption process. However, there is a limit to the amount of MB dye that can be adsorbed onto the surface area. Once all available surface sites are occupied, further increments in the adsorbent material do not increase adsorption capacity. Secondly, as the quantity of adsorbent material rises, there is heightened competition among the adsorbent particles to occupy the limited adsorption sites. This intensified competition necessitates MB dye molecules to compete with each other to bond with the restricted number of active sites on the adsorbent surface, thereby diminishing the overall adsorption capacity and hastening saturation. Thirdly, in certain instances, increasing the quantity of adsorbent material can elongate the diffusion path for MB molecules to reach the active sites on the adsorbent surface. Lengthier diffusion paths can decelerate the rate at which MB dye molecules can access and bond with the adsorbent, leading to a swifter saturation of the adsorption sites. Lastly, as the ratio of adsorbent material increases, the system may reach a juncture where mass transfer limitations become significant. This implies that the rate at which MB dye molecules can be conveyed from the bulk solution to the adsorbent surface becomes a constraining factor. Consequently, the adsorption capacity saturates more rapidly as the rate of adsorption fails to match the rate at which MB dye molecules approach the adsorbent surface^27,71^.

### GABS dose’s impact on adsorption capacity at different starting MB dye concentrations

**Figure 9:**
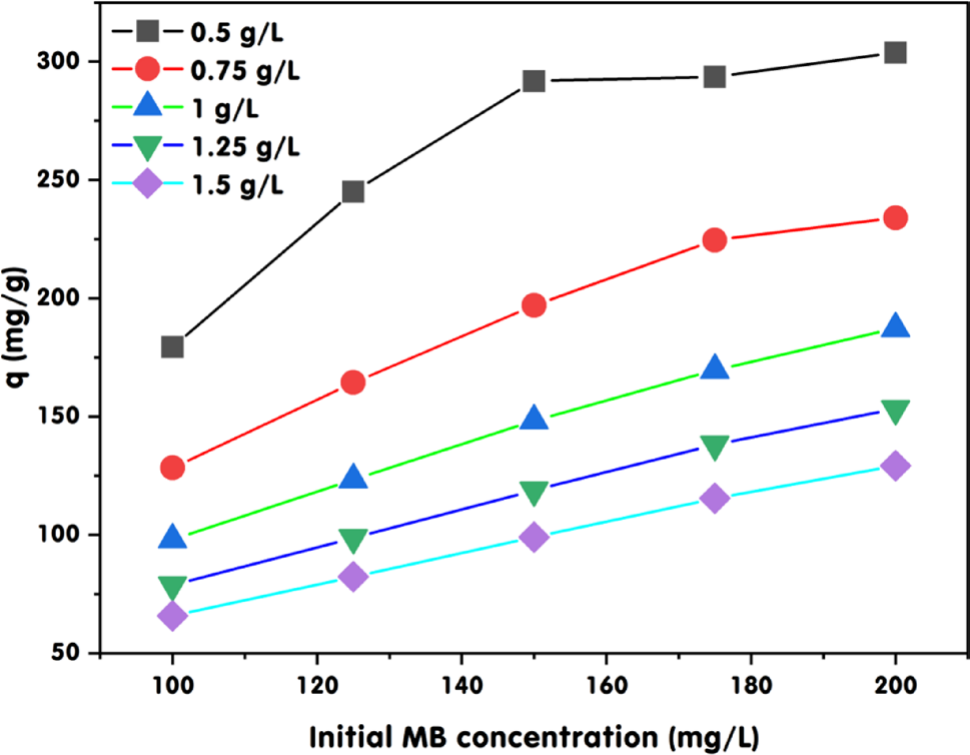
Impact of GABS dose on MB adsorption. The experimental conditions included a pH of 12, contact time of 180 min, and temperature of 25 ± 2 °C.

The data presented in Fig. 9 provides valuable insights into the relationship between the initial concentration of MB dye and its adsorption capacity on GABS at various adsorbent doses. This analysis offers significant illumination on how the dose of GABS influences the adsorption efficiency for different *C*_0_ of MB dye. Notably, at an initial MB dye concentration of 100 mg/L, the adsorption capacity of GABS at 0.5 g/L reached 179.41 mg/g, indicating a high efficiency of MB dye removal per unit of adsorbent. This notable adsorption capacity can be attributed to the abundance of active sites available for the lower concentration of MB dye, facilitating maximum interaction between MB dye molecules and GABS. However, as the *C*_0_ of MB dye increased to 125, 150, 175, and 200 mg/L, the adsorption capacity of GABS at 0.5 g/L also increased, reaching a peak of 303.78 mg/g at 200 mg/L of MB. This trend suggests that a higher *C*_0_ of MB dye may enhance the driving force for mass transfer, overcoming any resistance to adsorption and leading to a higher adsorption capacity. Conversely, at higher GABS doses ranging from 0.75 to 1.5 g/L, the adsorption capacity generally decreased with increasing adsorbent dosage. For instance, at an initial MB dye concentration of 200 mg/L, the adsorption capacity decreased from 234.00 mg/g at 0.75 g/L to 129.23 mg/g at 1.5 g/L.

The observation that the aggregation of adsorbent particles at higher doses reduces the effective surface area and active sites for methylene blue (MB) dye adsorption is supported by several relevant studies. The diminished adsorption capacity observed with increased adsorbent dosages is primarily attributed to the aggregation and overlapping of adsorbent particles, reducing the overall available surface area for dye adsorption. In a similar vein, the surface area may be reduced and the diffusional route length may be increased when adsorbent particles aggregate at increasing masses^72^. As the initial concentration of MB dye increases, the adsorption capacities at higher doses reach a plateau, signifying the approach to the equilibrium capacity of the adsorbent ^73^. All of these findings lend credence to the idea that adsorbent particle aggregation at higher dosages can decrease the number of active sites and effective surface area available for MB adsorption, which in turn can affect the adsorption capacity. The data underscores the adsorbent dose’s critical role in determining GABS’s adsorption capacity. While lower doses of GABS are more efficient in adsorbing MB dye per gram, they may not be practical for treating higher concentrations of MB dye due to their lower total adsorption capacity. Conversely, higher doses of GABS may be necessary to treat larger volumes or higher concentrations of MB dye, despite their lower efficiency per unit mass.

### Adsorption kinetic of MB dye onto GABS

**Figure 10:**
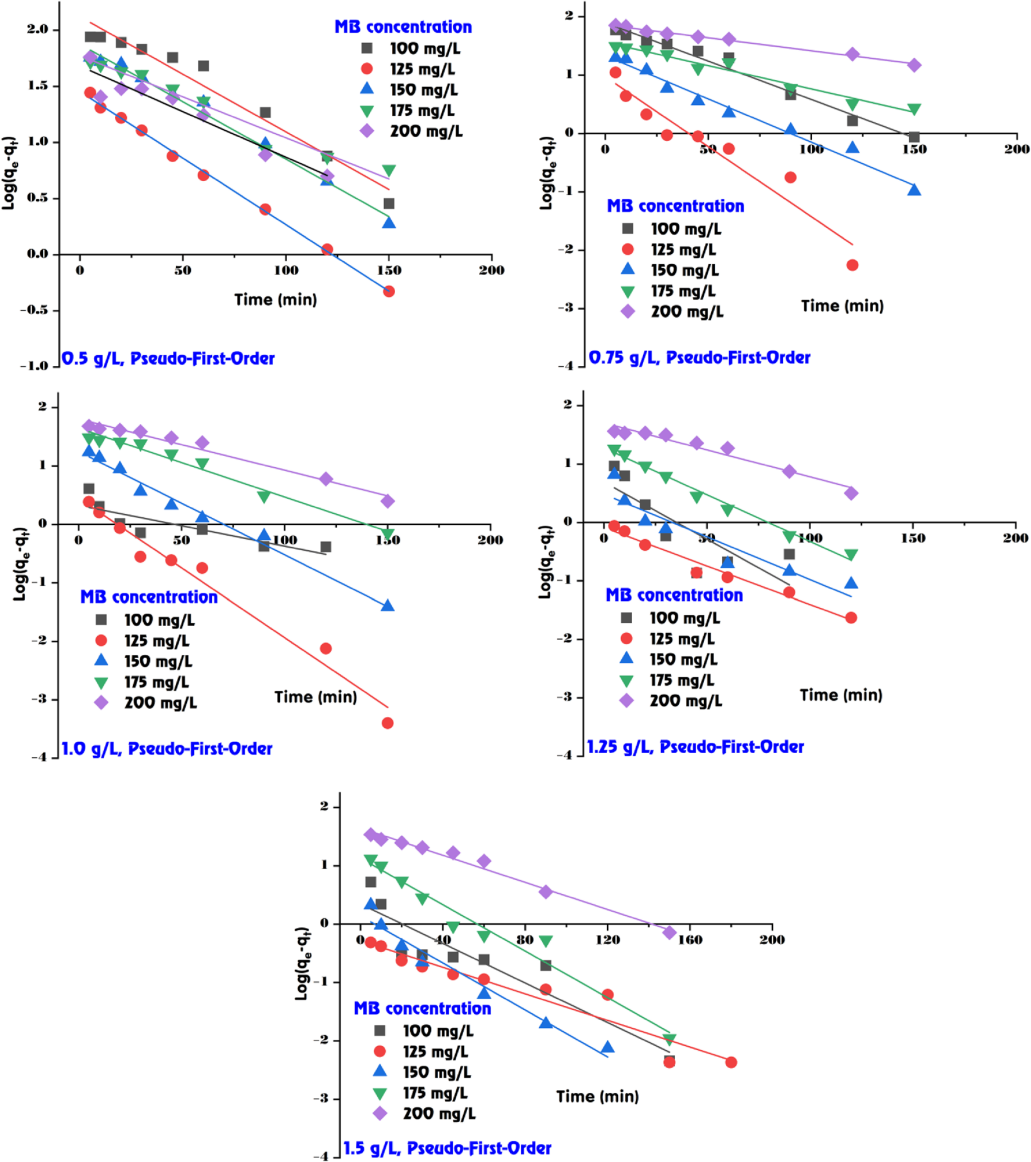
Pseudo-first-order model fitting for MB dye adsorption onto GABS.

**Figure 11:**
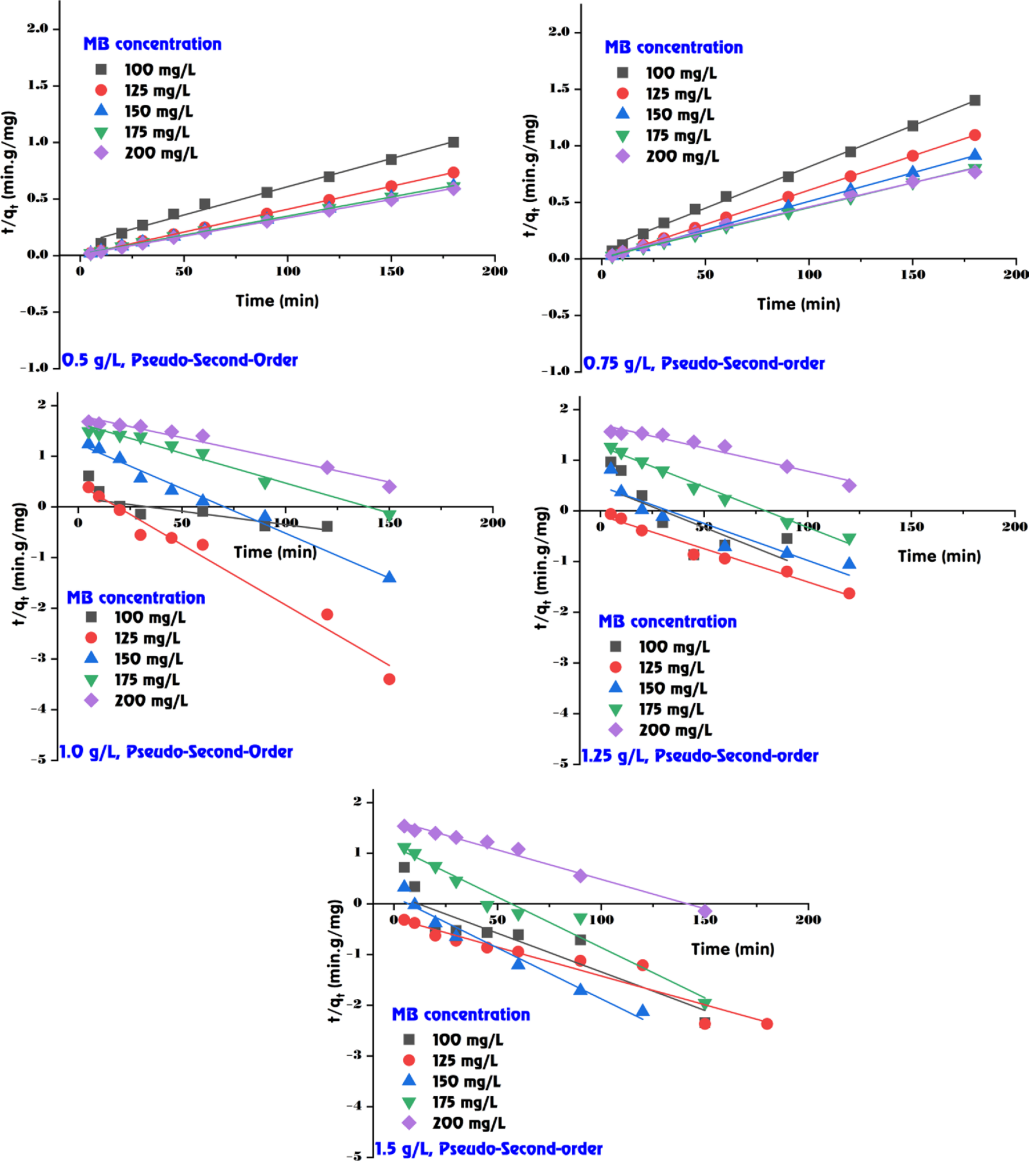
Pseudo-second-order model fitting for MB dye adsorption onto GABS.

The data presented in Table 2, Figs. 10 and 11 delineates the adsorption kinetics of MB dye on the GABS using the first-order and second-order kinetic models. These models are essential for comprehending the rate of adsorption and identifying the most suitable model for designing and predicting the behaviour of adsorption systems in practical applications. In the first-order kinetic model (Fig. 10), the adsorption rate constant (*k*_1_) and the calculated adsorption capacity (*q*_e,calc._) are compared against the experimental adsorption capacity (*q*_e,exp._). According to the first-order model, the proportion of occupied to vacant adsorption sites is determined by the occupancy rate of those sites. The values of *k*_1_ vary with both the dose of GABS and the initial concentration of MB dye. For instance, at a GABS dose of 0.5 g/L and an initial MB dye concentration of 100 mg/L, the *k*_1_ value is 23.72 × 10^–3^ with a *q*_e(calc.)_ of 132.07 mg/g, which is lower than the experimental value of 179.41 mg/g, indicating some deviation from the model. The *R*^2^ values for the first-order model, which show the model’s goodness of fit with the experimental data, are also presented (Table 2). While the *R*^2^ values are relatively high for all data sets, some are below 0.95, suggesting a less accurate model fit for those conditions.

**Table 2:** The calculated kinetic parameters of MB dye adsorption onto GABS.

Parameter			Pseudo-first-order			Pseudo-second-order			
GABS dose	Methylene blue (mg/L)	*q*_*e*_ (exp.)	*q*_*e*_ (calc.)	*k*_*1*_ × 10^3^	*R*^*2*^	*q*_*e*_ (calc.)	*k*_*2*_ × 10^3^	*h*	*R*^*2*^
0.5 g/L	100	179.41	132.07	23.72	0.963	200.00	0.23	9.12	0.991
	125	245.00	28.41	27.41	0.998	243.90	3.00	178.57	1.000
	150	291.81	74.92	23.49	0.990	294.12	0.86	74.63	0.999
	175	293.62	59.28	16.81	0.962	294.12	0.91	78.74	0.999
	200	303.78	48.02	18.65	0.918	303.03	1.20	109.89	1.000
0.75 g/L	100	128.33	79.05	30.17	0.980	135.14	0.74	13.57	0.997
	125	164.38	21.56	34.08	0.980	163.93	19.58	526.32	1.000
	150	196.97	36.49	18.42	0.966	200.00	4.63	185.19	1.000
	175	224.59	9.40	55.27	0.929	227.27	1.56	80.65	1.000
	200	234.00	72.88	10.13	0.990	232.56	0.59	31.95	0.995
1.0 g/L	100	98.00	2.13	16.12	0.715	98.04	41.62	400.00	1.000
	125	123.36	2.81	54.81	0.974	123.46	65.61	1000.00	1.000
	150	148.32	17.66	40.76	0.985	149.25	6.80	151.52	1.000
	175	169.66	44.60	27.18	0.975	172.41	1.53	45.45	0.999
	200	187.29	64.09	20.27	0.972	192.31	0.78	28.90	0.998
1.25 g/L	100	78.79	4.92	44.91	0.654	78.74	22.72	140.85	1.000
	125	98.74	1.22	30.17	0.971	99.01	127.51	1250.00	1.000
	150	118.85	3.05	33.62	0.870	119.05	37.14	526.32	1.000
	175	138.21	18.88	36.85	0.986	138.89	5.70	109.89	1.000
	200	153.27	50.65	21.19	0.951	156.25	1.03	25.19	0.998
1.5 g/L	100	65.74	2.23	38.92	0.844	65.79	50.23	217.39	1.000
	125	82.33	0.52	26.25	0.917	82.64	183.01	1250.00	1.000
	150	99.05	1.38	46.29	0.964	99.01	113.34	1111.11	1.000
	175	115.46	13.49	45.83	0.961	116.28	10.13	136.99	1.000
	200	129.23	44.38	26.71	0.982	133.33	1.45	25.84	0.999

The second-order kinetic model, on the other hand, frequently offers a better match for chemisorption processes as it posits that the adsorption rate is dependent on the availability of the adsorbate and adsorption sites (Fig. 11). The calculated *q*_e_ values are in closer agreement with the experimental data across most GABS concentrations, as evidenced by consistently high *R*^2^ values, indicating an excellent fit. The initial rate of adsorption (*h*), which can be interpreted as a combined effect of the rate constant *k*_2_ and the square of the equilibrium capacity, is also provided for the second-order model. These *h* values are notably high at lower GABS concentrations, especially at higher initial MB dye concentrations, suggesting a more efficient adsorption process under these conditions. The data indicates that the second-order kinetic model provides a better fit to the experimental data than the first-order model, particularly at lower GABS concentrations where the *R*^2^ values are perfect or near-perfect (1.000 or close to it), suggesting that the second-order kinetics are more appropriate for describing the adsorption behaviour of MB dye onto GABS. For instance, at a GABS dose of 0.5 g/L, the second-order model fits perfectly for initial MB dye concentrations of 125, 150, 175, and 200 mg/L with *R*^2^ values of 1.000, while the first-order model fits well but with slightly less congruence. At higher GABS doses, such as 1.5 g/L, the second-order model still provides a strong fit, although the first-order *R*^2^ values suggest a reasonable model agreement.

The kinetic analysis of the adsorption of MB onto GABS suggests that second-order kinetics closely describe the process across various adsorbent doses and initial MB dye concentrations, providing valuable insights for designing and optimizing adsorption systems for MB dye removal from wastewater. The second-order model demonstrates a firm fit, aligning with the first-order *R*^2^ values, indicating a reasonable model agreement. This finding is consistent with previous research that found the second-order kinetic model suitable for describing adsorption processes, as it provides essential information about the initial extraction process and the mechanism occurring in the final extraction stage^74^. Furthermore, the literature supports the use of second-order kinetics for modeling and predicting the behavior of adsorption systems under various operating conditions, emphasizing this model’s applicability in understanding and optimizing adsorption processes^75^.

### Adsorption isotherm of MB dye onto GABS

**Figure 12:**
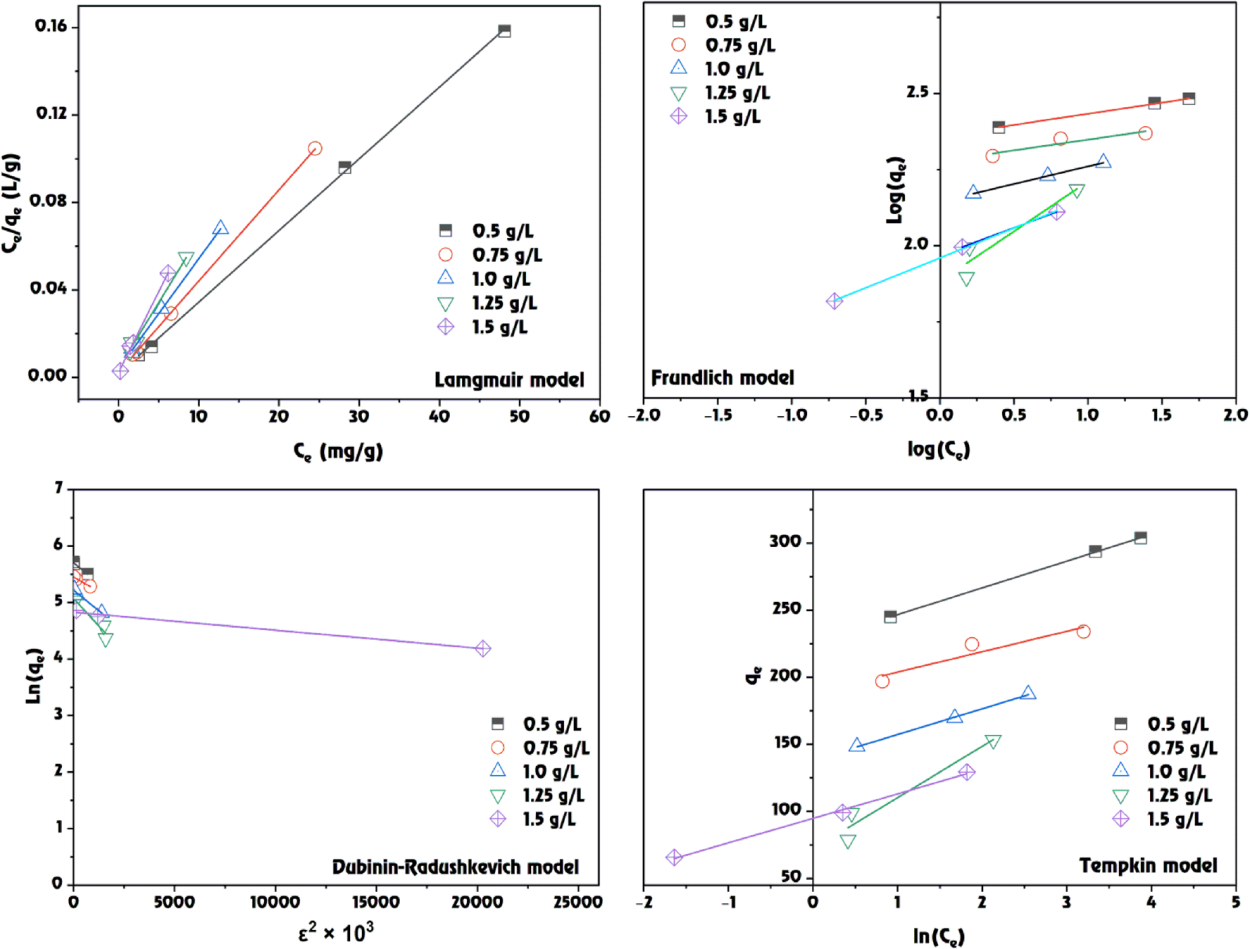
Isotherm model fitting for MB dye adsorption onto GABS.

The study presented in Table 3 outlines the application of various isotherm models to describe the adsorption of MB dye on the GABS at different concentrations (Fig. 12). Isotherm models are essential for understanding the interaction between adsorbates and adsorbents and are crucial in designing adsorption systems.

**Table 3:** The calculated isotherm and correlation constant parameters of MB dye adsorption onto GABS.

Isotherm	Isotherm	GABS dose				
Model	Parameters	0.5 g/L	0.75 g/L	1.0 g/L	1.25 g/L	1.5 g/L
Langmuir	*Q*_*m*_ (mg/g)	303.03	238.10	200.00	166.67	135.14
	*K*_*a*_	0.52	0.57	0.82	0.72	0.34
	*R*^*2*^	1.000	1.000	0.999	0.993	0.997
Freundlich	*1/n*	0.07	0.07	0.12	0.33	0.20
	*K*_*F*_ (mg^1–1/n^L^1/n^g^–1^)	229.19	189.63	139.77	76.35	91.20
	*R*^*2*^	1.000	0.879	1.000	0.903	0.999
Tempkin	*A*_*T*_	1.14E + 01	1.24E + 01	7.19E + 00	1.88E + 00	5.19E + 00
	*B*_*T*_	19.936	15.228	19.214	38.257	18.268
	*b*_*T*_	1.24E + 02	1.63E + 02	1.29E + 02	6.48E + 01	1.36E + 02
	*R*^*2*^	0.9999	0.8891	0.9992	0.944	0.9966
Dubinin–Radushkevich	*Q*_*m*_ (mol kg^–1^)	299.02	232.57	184.23	159.02	124.87
	*K* × 10^6^ (mol kJ^–1^)^2^	0.30	0.20	0.30	0.40	0.03
	*E* (kJ mol^–1^)	1.29E + 00	1.58E + 00	1.29E + 00	1.12E + 00	4.08E + 00
	*R*^*2*^	0.981	0.990	0.983	0.926	0.988

*Langmuir isotherm model* the maximum adsorption capacity (*Q*_m_) decreases with increasing GABS dose, indicating that lower doses of GABS have a higher capacity for adsorbing MB dye. The adsorption constant (*K*_a_) varies across different GABS doses, reflecting the strength of the adsorption bond. Assuming monolayer adsorption on a homogeneous surface with no interaction between adsorbed molecules, the *R*^2^ values are about 1.000 for all GABS dosages, showing a good match to the Langmuir model.

*Freundlich isotherm model* the heterogeneity factor (1/*n*) provides insight into the adsorption intensity or surface heterogeneity. Lower values suggest more homogeneous adsorption intensity. The Freundlich adsorption capacity (*K*_F_) decreases with increasing the GABS dose, implying reduced adsorption efficiency at higher adsorbent doses. The *R*^2^ values suggest that the Freundlich model fits well for most concentrations, although the fit is not as strong for 0.75 g/L.

*Tempkin isotherm model* the Tempkin isotherm constant (*A*_T_) decreases notably as the GABS dose increases, indicating a potential reduction in adsorption capacity. The Tempkin constants (*B*_T_ and *b*_T_) show variation with the GABS concentration, implying changes in the adsorption heat and surface coverage. The *R*^2^ values indicate a good fit for the Tempkin model, except for 0.75 g/L, where the fit is less satisfactory.

*Dubinin–Radushkevich (D–R) isotherm model* the theoretical saturation capacity (*Q*_m_) decreases with increasing the GABS dose, consistent with the Langmuir model. The D–R constant (*K*) suggests the nature of the adsorption process, with variations indicating differences in adsorption energy. The mean free energy (*E*) of adsorption provides insights into the adsorption type; energies less than 8 kJ/mol typically indicate physical adsorption. The *R*^2^ values suggest that the D–R model fits reasonably well across all concentrations. Therefore, the adsorption of MB dye onto the GABS is best described by the Langmuir model, suggesting homogenous adsorption onto a monolayer surface. However, the Freundlich model’s applicability across most concentrations indicates that adsorption also occurs on a heterogeneous surface with varying energies. The Tempkin model points to a decreasing heat of adsorption with increasing GABS concentration, and the D–R model suggests that the adsorption process is primarily physical. These models and their parameters can be invaluable in optimizing the design and operation of adsorption systems for MB dye removal using the GABS. The selection of the best-fit isotherm model is crucial for predicting the behaviour of the adsorption process under various conditions. It is evident that while the Langmuir model provides a very consistent fit, the relevance of the Freundlich and D–R models at different GABS doses highlights the complexity of the adsorption process, which may not be perfectly monolayer or homogeneous. These insights underline the necessity to consider multiple isotherm models for a comprehensive understanding of the adsorption mechanisms, significantly when scaling up from laboratory to industrial applications. The pseudo-second-order kinetic model has been found to provide a good correlation for the adsorption of MB onto various adsorbents, including clay, activated carbon, and biochar-coupled magnetic material^76–78^. Besides, the Langmuir model has shown a consistent fit for the adsorption of MB onto various materials, suggesting monolayer adsorption, while the Freundlich model has also been relevant at different concentrations, indicating heterogeneous surface energies^79,80^.

### Intraparticle diffusion and film diffusion model

**Figure 13:**
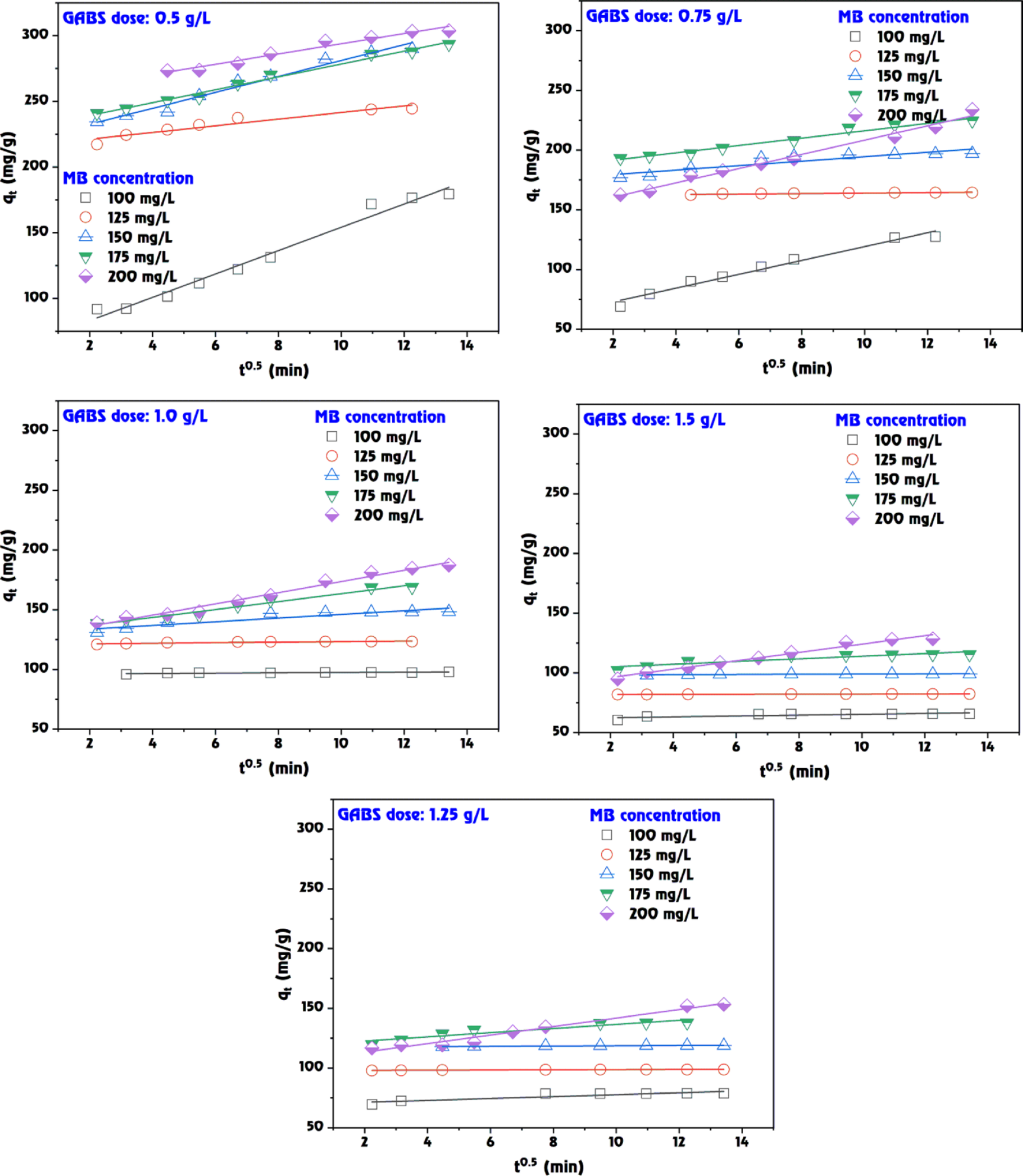
Intraparticle diffusion model fitting for MB dye adsorption onto GABS.

**Figure 14:**
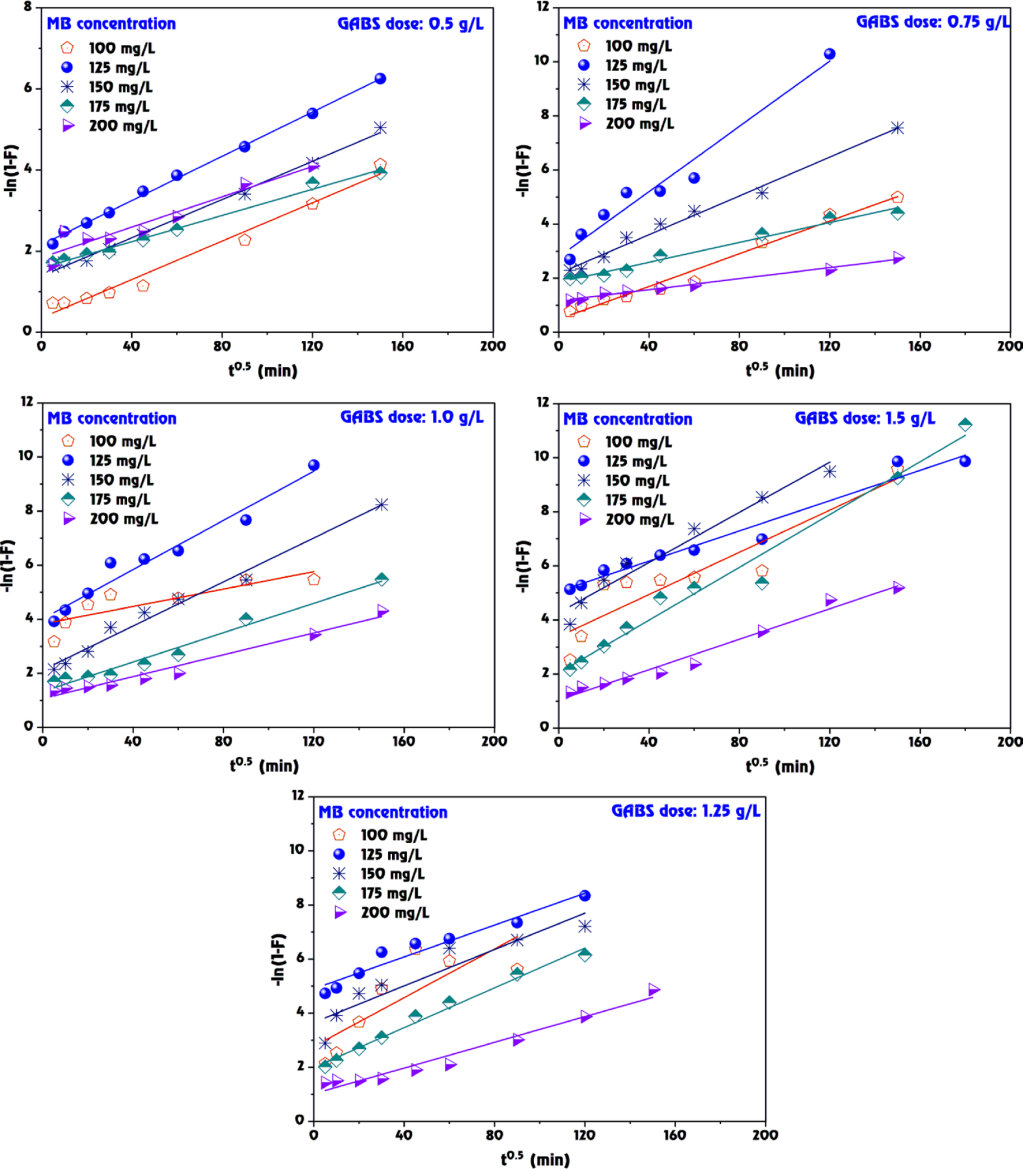
Film diffusion model fitting for MB dye adsorption onto GABS.

The data presents a comparative analysis of the intraparticle and film diffusion kinetics for the adsorption of MB dye on the GABS at various doses (Table 4, Figs. 13, 14). These models are crucial for characterizing the mass transfer mechanisms during the adsorption process^81^.

**Table 4:** The intended parameters for intraparticle and film diffusion models and correlation at MB dye adsorption onto GABS.

GABS dose (g/L)	Methylene Blue (mg/L)	Intraparticle diffusion			Film diffusion		
		*K*_*dif*_	*C*	*R*^*2*^	*K*_*FD*_	*C*	*R*^*2*^
0.5	100	8.881	65.38	0.981	0.0236	0.356	0.978
	125	2.533	216.21	0.915	0.0273	2.155	0.998
	150	6.063	220.47	0.974	0.0236	1.387	0.995
	175	4.904	229.40	0.989	0.0161	1.589	0.991
	200	3.886	254.98	0.961	0.0187	1.845	0.918
0.75	100	5.800	61.24	0.976	0.0302	0.485	0.980
	125	0.201	162.02	0.777	0.0603	2.791	0.963
	150	1.872	175.79	0.830	0.0358	2.175	0.989
	175	3.071	185.45	0.974	0.0184	1.854	0.980
	200	5.961	148.77	0.986	0.0102	1.166	0.990
1.0	100	0.136	96.11	0.719	0.0161	3.830	0.715
	125	0.218	121.13	0.777	0.0473	3.951	0.984
	150	1.536	130.76	0.855	0.0407	2.128	0.985
	175	3.317	130.32	0.972	0.0271	1.336	0.975
	200	4.689	126.77	0.982	0.0202	1.072	0.972
1.25	100	0.797	69.71	0.820	0.0450	2.774	0.654
	125	0.076	97.86	0.870	0.0294	4.902	0.946
	150	0.113	117.50	0.841	0.0336	3.661	0.870
	175	1.737	119.20	0.891	0.0368	1.990	0.986
	200	3.571	106.15	0.973	0.0237	1.023	0.965
1.5	100	0.369	61.65	0.651	0.0390	3.386	0.844
	125	0.042	81.82	0.904	0.0280	5.058	0.965
	150	0.074	98.22	0.676	0.0464	4.271	0.964
	175	1.110	102.84	0.820	0.0488	2.041	0.977
	200	3.508	89.05	0.976	0.0281	1.040	0.978

*Intraparticle diffusion* the intraparticle diffusion rate constant (*K*_dif_) signifies the rate at which the MB dye moves through the boundary layer to the adsorbent’s surface (Fig. 13). The *K*_dif_ values exhibit variability with both the GABS dose and MB dye concentration. For instance, at the GABS dose of 0.5 g/L and an initial MB dye concentration of 100 mg/L, *K*_dif_ is 8.881, which decreases to 3.886 as the MB dye concentration increases to 200 mg/L. The intercept (C) represents the boundary layer thickness, with higher values suggesting a more significant contribution of surface diffusion in adsorption. For example, C increases from 65.38 to 254.98 as the MB dye concentration increases from 100 to 200 mg/L at a GABS dose of 0.5 g/L. The coefficient of determination (*R*^2^) suggests a good fit for the intraparticle diffusion model, with values close to 1.0000, indicating that the model reliably represents the diffusion process across most conditions.

*Film diffusion* the film diffusion coefficient (*K*_FD_) reflects the mass transfer rate of MB dye across the liquid film surrounding the GABS particles (Fig. 14). At the GABS dose of 0.5 g/L, *K*_FD_ ranges from 0.0236 to 0.0187 as the MB dye concentration increases from 100 to 200 mg/L, indicating that the film diffusion rate may decrease with higher MB dye concentrations. Similar to the intraparticle diffusion model, the *C* value for film diffusion suggests the impact of the external mass transfer resistance. For instance, at 0.5 g/L GABS dose, the *C* value varies significantly, reaching up to 2.155 for an MB dye concentration of 125 mg/L. The *R*^2^ values are high across all conditions, particularly for the film diffusion model, demonstrating an excellent fit and suggesting that film diffusion is a significant factor influencing the adsorption kinetics of MB dye onto the GABS. At a GABS dose of 1.5 g/L, the film diffusion model shows an *R*^2^ of 0.977 for an MB dye concentration of 175 mg/L, indicating that film diffusion is a significant step in controlling adsorption. The intraparticle diffusion *R*^2^ is lower (0.820) at the same conditions, implying that while intraparticle diffusion contributes to the process, the film diffusion could be the rate-limiting step.

The film diffusion seems more consistent with higher *R*^2^ values across all doses and concentrations, indicating it may often be the controlling mechanism (Fig. 14). Besides, the intraparticle diffusion cannot be neglected, especially at lower GABS doses and MB dye concentrations, where its contribution is significant. The film and intraparticle diffusion process plays an essential role in adsorption systems.

### Comparison of results with reported literature

A selection of earlier research on the removal of MB dye ions from aquatic medium is shown in Table 5. According to the maximum adsorption capacity (*Q*_m_) reported in this Table, at room temperature, the GABS adsorbent has the highest *Q*_m_ for MB dye among the literature reported in Table 5. For the elimination of MB dye ions at a given concentration of 0.5 g/L of GABS adsorbent, these values were 303.03 mg/g. It is clear from this comparison that the GABS made from green algae was a highly effective adsorbent for taking out MB dye from aqueous solutions.

**Table 5:** The highest amount (*Q*_m_, mg/g) of MB dye that can be adsorbed onto several documented adsorbent materials.

Biosorbents	*Q*_m_ (mg g^–1^)	References
GABS	303.03	This study
Marine alga *U. lactuca*	40.2	^82^
Cedar sawdust	142.36	^83^
Crushed brick	96.61	^83^
Sugar beet pulp	244.76	^84^
Watermelon rind	231.48	^85^
Inactivated rosemary plant	153.17	^86^
Activated carbon from rosemary plant	110.67	^86^
Corn *Stigmata*	106.3	^87^
Orange peel	280	^88^
Jute stick charcoal	29.32	^89^
*Azolla pinnata*	80.6	^90^
*Shorea spp.*	37.8	^91^

### Regeneration study

**Figure 15:**
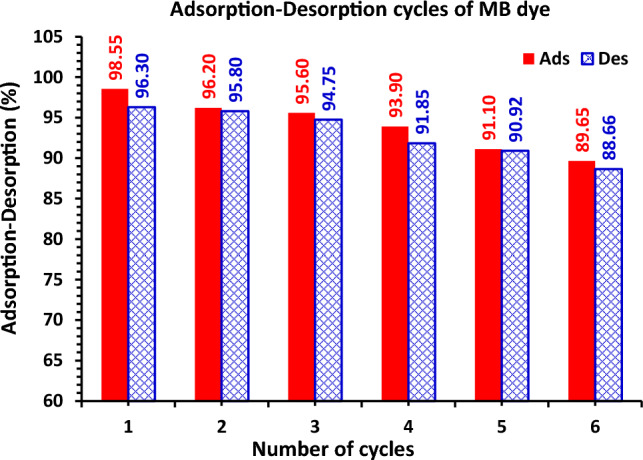
Renewal study of MB dye adsorption–desorption by GABS adsorbent using dye *C*_0_ (100 ppm) and 1.5 g/L GABS dose at room temperature.

Desorption studies of the MB dye from the GABS adsorbent were carried out using 0.1 M NaOH and HCl followed by ethanol as an elution media to examine the viability and reusability of the adsorbent for the adsorption of MB dye. In this investigation, when the regeneration cycles increased, the proportion of dye desorption decreased (Fig. 15). Using the regenerated GABS, six cycles of adsorption/desorption have been investigated. Throughout the cycles, the changes in adsorption and desorption were mostly almost constant^12,65^. However, after six cycles, it decreased by about 9.9% for MB dye. It advises using GABS as a long-lasting method of removing MB colour from water (Fig. 15).

### MB dye adsorption mechanism using GABS

**Figure 16:**
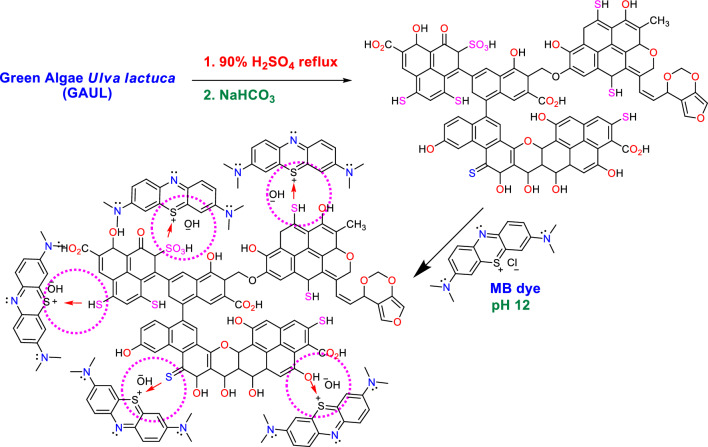
The most likely process by which the produced GABS adsorbent absorbs the MB dye.

The most plausible process by which GABS absorbed the MB dye ions is described in Fig. 16. After the green algae, *U. lactuca* source material has been dehydrated by 90% using H_2_SO_4_. FTIR examination revealed that a range of functional groups, such as C=O, CO_2_H, C–O–C, O–H, SO_3_H, C–S, SH, and SO groups, formed on the adsorbent’s surface (GABS). due to the positive charge on the MB dye in the presence of OH to stabilise the newly formed bond, as well as the electrostatic contact between the sulphur and oxygen lone pair on the GABS surface. Electrostatic interaction may be used to carry out the MB dye ions’ adsorption procedure in a high basic media (pH 12.0).

In a strong basic environment, the surface of GABS picks up a positive charge on the MB dye, which attracts negatively charged OH^25,92^. Furthermore, dye molecules are more soluble at a basic pH, which makes it easier for them to adhere to the adsorption sites and diffuse through the BABS’s pores. The most significant process is the ionizable organic molecules’ adsorption to the positively or negatively charged surface of the biochar through electrostatic contact^93–104^.

Moreover, the pH of the solution affects the organic pollutants’ ability to adsorb in industrial effluent^93^. The application of biochar made from food waste for the adsorption of textile colours in wastewater was investigated by Parshetti et al.^105^. They discovered that dye adsorption was boosted by an alkaline pH. The significant interaction between the negatively charged sites on the surface of the biochar and the positively charged dyes explained it. According to Tsai and Chen^106^ and Xu et al.^107^, the ability of biochar to absorb materials is affected by pH. The pH of the solution therefore influences the charged sites and the ability of organic and inorganic contaminants from industrial effluent to adsorb on biochar.

## Conclusion

The study on the adsorption efficiency of MB dye from aqueous solutions using the GABS offers valuable insights into the nuanced dynamics of MB dye adsorption onto the GABS. The findings highlight vital observations, including the optimal adsorption capacity of 303.78 mg/g at the GABS dose of 0.5 g/L and the 200 mg/L MB dye concentration. Furthermore, the Langmuir isotherm model consistently fits the adsorption data, indicating monolayer adsorption on a homogeneous surface, with the highest adsorption capacity (*Q*_m_) of 303.03 mg/g. The applicability of the Freundlich and Tempkin models across different GABS doses underscores the complexity of the adsorption process, emphasizing the need for a multifaceted understanding of the adsorption phenomena, particularly when scaling from laboratory to industrial applications. Moreover, the study demonstrates that the pseudo-second-order kinetic model offers a better correlation than the first-order model for the adsorption data, highlighting its suitability for predicting the system’s behaviour under various conditions. These detailed insights are instrumental for designing and optimizing industrial-scale adsorption systems for effective MB dye removal from wastewater. The findings advocate for a tailored approach based on specific operational parameters, emphasizing the importance of considering various kinetic and isotherm models to understand the adsorption process comprehensively. The study’s comprehensive analysis of the adsorption dynamics of MB dye onto GABS contributes to the existing body of knowledge on wastewater treatment technologies. The insights provided by this research can inform the development of efficient and sustainable water treatment solutions, particularly in industrial-scale applications. By elucidating the nuanced dynamics of MB dye adsorption onto GABS and emphasizing the significance of considering various operational parameters, the study offers a valuable foundation for further research and development in adsorption-based water treatment technologies.

## Data Availability

Should any raw data files be needed in another format they are available from the corresponding author upon reasonable request.
